# The Use of Biologic Modifiers as a Bridge to Hematopoietic Cell Transplantation in Primary Immune Regulatory Disorders

**DOI:** 10.3389/fimmu.2021.692219

**Published:** 2021-06-24

**Authors:** Danielle E. Arnold, Deepak Chellapandian, Jennifer W. Leiding

**Affiliations:** ^1^ National Cancer Institute, National Institutes of Health, Bethesda, MD, United States; ^2^ Center for Cell and Gene Therapy for Non-Malignant Conditions, Cancer and Blood Disorders Institute, Johns Hopkins All Children’s Hospital, St. Petersburg, FL, United States; ^3^ Division of Allergy and Immunology, Department of Pediatrics, University of South Florida, St. Petersburg, FL, United States

**Keywords:** PIRD, CTLA4, Pi3Kinase, STAT1, STAT3, Jakinib, Abatacept, Emapalumab

## Abstract

Recently, primary immune regulatory disorders have been described as a subset of inborn errors of immunity that are dominated by immune mediated pathology. As the pathophysiology of disease is elucidated, use of biologic modifiers have been increasingly used successfully to treat disease mediated clinical manifestations. Hematopoietic cell transplant (HCT) has also provided definitive therapy in several PIRDs. Although biologic modifiers have been largely successful at treating disease related manifestations, data are lacking regarding long term efficacy, safety, and their use as a bridge to HCT. This review highlights biologic modifiers in the treatment of several PIRDs and there use as a therapeutic bridge to HCT.

## Introduction

Advances in genetic testing have led to the discovery of many new immune disorders characterized by immune mediated pathology, coined primary immune regulatory disorders (PIRD). As the mechanisms of PIRDs are elucidated, biologic modifiers that alter the mechanism of disease have provided precision-based therapies successfully treating clinical disease manifestations. Hematopoietic cell transplant (HCT) has been successful in treating patients with PIRD as a definitive life-saving therapy in patients with severe disease. Although biologic modifiers have the ability to treat disease manifestations, data is lacking on whether they prevent the development of symptoms to begin with and what the long-term utility and safety profile of these agents will be. Further, what their role will be in patients undergoing HCT is unclear. There is growing evidence that when disease manifestations of PIRD are controlled or in remission, outcomes of HCT are improved. To that end, biologic modifiers have been successful in controlling life threatening disease manifestations of PIRD and are considered a bridge to more definitive therapies such as HCT in patients with severe disease manifestations.

The purpose of this article is to review PIRDs in which biological modifiers have been successful in the control of disease manifestations. Also discussed are outcomes of HCT and evidence supporting the use of biological modifiers to induce remission or control of immune dysregulation, and how that control affects HCT outcomes.

## Hemophagocytic Lymphohistiocytosis and Hyperinflammatory Disorders

Hemophagocytic lymphohistiocytosis (HLH) is a life-threatening immune dysregulation disorder caused by impairment of the cytotoxic function of NK cells and CD8+ T-cells, thereby resulting in uncontrolled systemic inflammation, hypercytokinemia and tissue damage. HLH can be either primary (also called familial) or secondary. Primary HLH can be caused by defects in genes such as *PRF1*, *UNC13D*, *STX11*, *STXBP2*, *RAB27A*, *LYST*, and *AP3B1*, all of which are essential for NK cell or T cell cytotoxicity (1–7). X-linked lymphoproliferative disorders (XLP) 1 and XLP 2 are two genetic X-linked diseases that predispose to HLH mostly in the context of Epstein Barr virus (EBV) infection (8, 9). Heterozygous mutation of *CDC42* (10) and *NLRC4* (11) cause autoinflammatory conditions that mimic HLH, characterized by excessive elevation of free interleukin (IL)-18 and IL-1β. HLH can be a symptom of other primary immunodysregulatory disorders or can be secondary in immunocompetent patients with concurrent infection, rheumatic disease, or malignancy (12).

HLH is a clinical spectrum characterized by fever, cytopenias, hepatosplenomegaly, rash, elevated transaminases, coagulopathy and central nervous system (CNS) involvement.

Diagnosis requires either a genetic confirmation or fulfillment of 5 of 8 diagnostic criteria: fever; splenomegaly; cytopenias in at least 2 lineages; hypertriglyceridemia and/or hypofibrinogenemia; hemophagocytosis observed in the bone marrow, spleen, liver, lymph nodes or other tissues; low or absent NK cell activity; elevated ferritin; and elevated levels of soluble IL-2 receptor (sIL-2R) (13). The established diagnostic criteria have limitations, as some patients may only present with incomplete or atypical manifestations.

HLH is universally fatal if left untreated. The key therapeutic strategies in management include: induction of remission and control of hyperinflammation, elimination of trigger, and definitive therapy of the underlying or primary condition. Induction of remission and control of hyperinflammation is achieved using potent immunosuppressive and chemotherapeutic agents. A regimen containing etoposide and dexamethasone derived from HLH-94 and HLH-2004 study protocols has been widely used as frontline therapy for HLH (14, 15). The rate of remission induction with this regimen was 71%, and 5-year probability for survival following hematopoietic cell transplant (HCT) was 54% ± 6%. Upfront addition of cyclosporine to dexamethasone and etoposide did not improve the outcome of patients in the HLH-2004 study (15).

In a single center study of 38 patients, a chemotherapy sparing regimen consisting of steroids, anti-thymocyte globulin (ATG), and cyclosporine showed a promising remission rate of 70%, however there was higher rate of relapses prior to HCT (16). Alemtuzumab, a humanized monoclonal anti-CD52 antibody, in combination with methylprednisolone and cyclosporine induced partial response in 64% of treated patients (17). A clinical trial evaluating alemtuzumab as first line treatment of primary HLH is currently underway (NCT02472054). In patients that are refractory to etoposide-containing regimens, alemtuzumab could be considered salvage therapy to induce remission prior to proceeding to HCT (17).

Interferon gamma (IFNγ) has been shown in several *in vitro* and *in vivo* studies as a major driver of pathological inflammation in HLH. As such, blockade of IFNγ can interrupt the inflammatory cascade and restore immune homeostasis (18). Emapalumab, a recombinant human monoclonal antibody directed against IFNγ, has been recently approved for use in patients with primary HLH that is refractory, recurrent, progressive or intolerant to conventional HLH therapy (19, 20). The safety and efficacy of emapalumab was studied in a pivotal phase II/III clinical trial (NCT01818492) in patients ≤ 18 years of age with primary HLH showing an overall response rate of 64.7% and overall survival to HCT of 79%. Treatment with emapalumab was associated with decreased circulating levels of IFNγ, and its inducible chemokine CXCL9, correlating with improvement in laboratory parameters and decreasing levels of pro-inflammatory cytokines (21). Emapalumab is also being tested in other secondary forms of HLH with characteristic IFNγ signature including systemic juvenile arthritis developing macrophage activation syndrome (NCT03311854).

The Janus Kinase (JAK)/Signal Transducer and Activation of Transcription (STAT) pathway lies downstream of IFNγ and several other pro-inflammatory cytokines that are elevated in HLH and could represent an attractive therapeutic target to abrogate the signaling of multiple cytokine pathways at the same time. JAK/STAT blockade using ruxolitinib, a JAK1/2 inhibitor, has been shown to dampen HLH disease manifestations including CNS involvement in the *Prf1^-/-^* murine model of HLH (22, 23). Preliminary results from a single center pilot trial have shown good response in secondary HLH (24). A phase Ib/II trial is currently evaluating the efficacy and tolerability of ruxolitinib in combination with dexamethasone and etoposide as a frontline therapy in newly diagnosed HLH or as salvage therapy in relapsed or refractory HLH (NCT04551131).

Free IL-18, a cytokine released by activated macrophages and not bound to its binding protein (IL-18 BP), is an interesting target in certain hyperinflammatory conditions. Several studies have reported elevated levels of free IL-18 in the serum of patients with primary and secondary HLH and levels have correlated with HLH symptom progression (25). A phase III, double-blinded placebo-controlled clinical trial using IL-18 BP (tadekinig alpha) is currently evaluating the efficacy and tolerability of IL-18BP in IL-18 driven autoinflammatory conditions including *NLRC4* associated hyperinflammation and XIAP deficiency (NCT03113760). Other pro-inflammatory cytokines elevated in the HLH include IL-1, IL-6 and TNF-alpha. Targeted monoclonal antibodies and small molecule inhibitors targeting these cytokines have been used with mixed success (26–29).

Although primary HLH can be controlled by cell-based and cytokine targeted therapy, HCT serves as the only optimal therapy in ultimately preventing recurrences. HCT for HLH previously had dismal results, but outcomes have improved substantially with the control of hyperinflammation prior to HCT (30, 31). In the largest prospective HLH trial, uncontrolled active disease at time of HCT was associated with higher transplant related mortality and graft failure (31). Heightened IFNγ has correlated with poor engraftment rates and worse outcomes in a murine model (18) and more recently, serum levels of IFNγ and CXCL9 have been found to be significantly higher in patients with post-HCT graft failure (32) indicating control of IFNγ-driven hyperinflammation prior to HCT is imperative and directly correlates with rates of graft failure and survival (31, 32). In the same study, 2 of 3 patients with HLH and graft failure had successful donor engraftment when concurrently treated with emapalumab on a compassionate use basis while being re-transplanted with the same HLA-haploidentical donors respectively (32). These clinical observations further support the need to blunt IFNγ-driven inflammation prior to HCT in patients with HLH. Pre-emptive use of emapalumab is being considered for clinical trial post allogeneic HCTs in which there is a high risk of graft failure. HCT remains the only curative option for various primary immunodeficiency disorders, including XLP1 (33) and XLP2 (34), however the role of HCT in NLRC4 deficiency remains controversial.

## Cytotoxic T Lymphocyte Antigen-4 Haploinsufficiency and Lipopolysaccharide Responsive Beige-Like Anchor Deficiency

**Figure 1:**
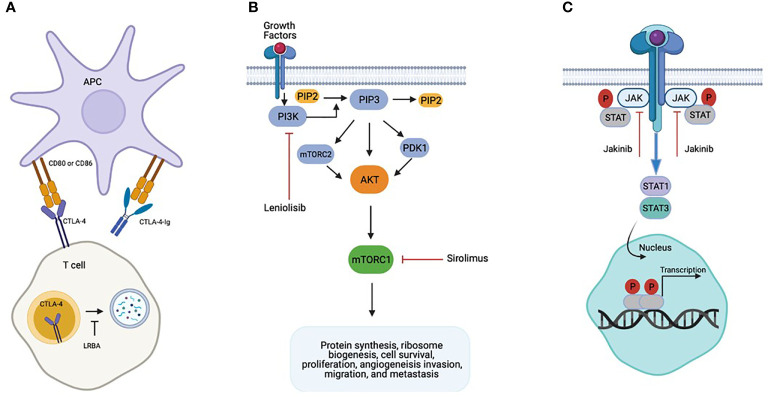
Biology of Precision Based Therapies. **(A)** CTLA4-Ig binds to CD80/86 effectively replacing the non-functional CTLA-4 protein. **(B)** Gain of Function mutations in PIK3CD or PIK3RI result in Activated PI3K d Syndrome. Leniolisib inhibits PI3K activation directly and sirolimus inhibits downstream enhanced mTORC1 activity. **(C)** Gain of function mutations in STAT1 and STAT3 cause hyperactivation STAT1 and STAT3 respectively. Jakinibs are direct inhibitors of the JAK/STAT pathway.

Pathogenic variants in cytotoxic T-lymphocyte-associated protein four (CTLA-4) and lipopolysaccharide-responsive beige-like anchor (LRBA) protein have been recently described to be associated with complex immune dysregulation syndromes (35, 36). CTLA-4 is a homolog to the costimulatory surface protein CD28, which is expressed on the activated T cells including regulatory T cells (Tregs) and serves as a negative immune regulator crucial for maintaining self-tolerance and immune homeostasis (37) (**Figure 1A**). Lipopolysaccharide-responsive beige-like anchor (LRBA) protein deficiency, occurs due to complete loss of expression of LRBA protein resulting from biallelic mutations in *LRBA* (38, 39). LRBA is a ubiquitously expressed cytosolic protein that regulates the traffic of intracellular vesicles, and acts as a chaperon for CTLA-4 that prevents it from lysosomal degradation (**Figure 1A**). Absence of LRBA leads to decreased CTLA-4 expression, thereby resulting in defective Treg cell function causing immune dysregulation and autoimmunity (36).

CTLA-4 binds to the CD80 and CD86 ligands on antigen presenting cells (APCs) outcompeting CD28 mediated activating signals thereby downregulating immune response by inhibiting the APC mediated T cell activation. Patients with CTLA-4 haploinsufficiency present with clinical and laboratory findings consistent with common variable immunodeficiency (CVID), autoimmune cytopenias (AIC), lymphocytic infiltration of nonlymphoid organs, and malignancy. The immune phenotype can include lymphopenia of CD8+ T, B, and natural killer (NK) cells, proportional increase in CD4+T cells, and impaired response to immunizations. Reduction of class switched B cells with increased proportion of autoreactive CD21^low^ B cells have also been reported (35). LRBA deficiency was originally reported in 2012 in 4 consanguineous families who presented with early onset antibody deficiency and autoimmunity (40). Due to interaction with CTLA-4, there is partial resemblance in immunological and clinical presentation of these two conditions with presentations including hypogammaglobulinemia, recurrent infections, and autoimmunity such as enteropathy, AIC, and polyendocrinopathy (41, 42).

Rapamycin, a mechanistic target of rapamycin inhibitor (mTOR), inhibits the CD28 signaling pathway thereby decreasing T cell hyperactivity and immune dysregulation (43). Furthermore, rapamycin has been suggested to increase the number of Tregs. These effects coupled with a relatively benign side effect profile makes rapamycin an attractive therapeutic option in treatment of autoimmune manifestations in both CTLA-4 haploinsufficiency and LRBA deficiency. Treatment with rapamycin led to improvement in AIC, regression of lymphadenopathy and splenomegaly, and decreased consumption of immunoglobulin (44) (**Table 1**). Tesch et al. described the largest cohort of 76 LRBA deficient patients, 52 were treated with conventional immunomodulator therapies without undergoing HCT. Of 16 patients who were treated with rapamycin alone: enteropathy improved in 57%, parenchymal lung disease in 38%, and AIC in 63% (**Table 1**).

**Table 1:** Targeted therapy for CTLA-4 Haploinsufficiency, LRBA Deficiency, Activated PI3Kδ syndrome, and STAT1 or STAT3 Gain of Function.

Reference	N	Biologic	Indications for treatment	Response	Follow-up
**CTLA-4 Haploinsufficiency**						
Schwab et al. (44)	14	CTLA4 fusion protein (abatacept or belatacept)	Lymphoproliferation, Enteropathy, Interstitial lung disease, cytopenia	11/14 (79%) with clinical improvement 6 patients with CR of enteropathy 2 patients with PR of ILD 2 patients had additional IST discontinued after starting abatacept 6 patients had treatment discontinued- 3 proceeded to HCT, 2 EBV reactivation, 1 with severe respiratory tract infection	Not reported	
Schwab et al. (44)	13	Sirolimus	Lymphoproliferation, Enteropathy, Cytopenia	8/13 (62%) with clinical improvement- response included resolution of red cell aplasia, regression of lymphadenopathy and splenomegaly, reduction in Ig consumption. 3 patients with PR of enteropathy 2 patient had treatment discontinuation due to ineffectiveness	Not reported	
**LRBA Deficiency**						
Tesch et al. (45)	23	Abatacept	Lymphoproliferative, autoimmune or inflammatory disease	14/23 (61%) had significant reduction in immune dysregulation (IDDA) score and organ specific symptoms 10/14 (71%) with good general response with amelioration of all symptoms	400 patient mos (0.1-5yrs)	
Tesch et al. (45) Lo et al. (36)	16 3	Sirolimus Abatacept	Lymphoproliferative, autoimmune or inflammatory disease, neurological manifestation Presentation as CVID; Intractable enteritis and autoimmune or inflammatory disease	57% with amelioration of symptoms 38.5% with improvement in lung disease 37.5% with resolution of AIC 4/5 (80%) had improved neurological manifestations 29% with improvement in malabsorption and FTT 35% with fewer or less severe infections 3/3 (100%) improvement in abdominal symptoms, weight gain, resolution of arthritis and reduction in frequency of infections	Not reported 6 months (last reported)	
**Activated PI3Kδ Syndrome**						
Coulter et al. (46)	6	sirolimus	Lymphoproliferative, autoimmune or inflammatory disease	5/6 (83%) with clinical improvement/decrease in lymphoproliferation	Not reported	
Elkaim et al. (47)	6	sirolimus	Lymphoproliferation	2 patients with adequate follow-up had significant reduction in lymphoproliferation	Not reported	
Maccari et al. (48)	26	sirolimus	Lymphoproliferation (25) Bowel inflammation (15) Cytopenia (14)	8/25 (32%) CR and 11/25 (44%) PR lymphoproliferation 3/15 (20%) CR and 3/15 (20%) PR bowel inflammation 3/14 (21%) CR and 2/14 (14%) PR autoimmune cytopenia 8/8 (100%) of patients on steroids were able to discontinue steroids or have steroid dose reduced	Average time of therapy monitoring 1.6 years	
Rao et al. (49)	6	leniolisib	Recurrent sinopulmonary infections ± bronchiectasis (5) Cytopenia (3) Lymphoma (3)	6/6 (100%) with reduction in lymph node and spleen size 6/6 (100%) with improved energy levels and overall increased well being	12 weeks	
**STAT1 or STAT3 Gain of Function**						
Forbes et al. (50)	11	Jakinib (ruxolitinib or tofacitinib)	Autoimmune or immune dysregulation (16) Immune suppression prior to HCT (1) HLH (1) Chronic progressive infection (8)	14/17 (82%) with significant clinical improvement Two patients with TPN-dependent enteropathy became TPN independent Autoimmune cytopenia improved in 4 patients with STAT1 GOF and 4 patients with STAT3 GOF CMC resolved in all patients with STAT1 GOF	Median 8 mos (1-34 mos)	

CR, complete response; PR, partial response; ILD, interstitial lung disease; IST, immunosuppressive therapy; HCT, hematopoietic cell transplantation; EBV, Epstein Barr virus; Ig, immunoglobulin; IDDA, immune deficiency and dysregulation activity score; AIC, autoimmune cytopenia; FTT, failure to thrive; HLH, hemophagocytic lymphohistiocytosis; TPN, total parenteral nutrition; GOF, gain of function; CMC, chronic mucocutaneous candidiasis.

The functional interplay of LRBA and CTLA-4 in T cell homeostasis has provided the rationale to treat both conditions using abatacept. Abatacept and belatacept are fusion proteins that contain an extracellular domain of CTLA-4 and the Fc portion of human IgG1 (CTLA-4-Fc-IgG1) and serve as a replacement for the functional loss of CTLA-4 (**Figure 1A**). Fusion proteins like abatacept and belatacept have shown to be an effective targeted treatment to control immune dysregulation in CTLA-4 haploinsufficiency and LRBA deficient patients (51). Improvement or resolution of AIC, enteropathy, hepatitis, and lymphoproliferation have been reported (36, 44, 45, 52, 53). Treatment with abatacept also resulted in improvement in immunological phenotype as shown by increasing naive: effector T cell ratios, and improved functional antibody responses to polysaccharide vaccines (**Table 1**).

The use of abatacept and belatacept in CTLA-4 haploinsufficiency and LRBA deficiency appears to be a promising first line therapy to control manifestations of immune dysregulation, however, the lifelong use of this therapy is limited by increased susceptibility to infections and possible evolution of malignancies (54). HCT should be carefully considered as a possible definitive therapy especially in patients who do not respond to non-steroid targeted intervention as described above. HCT could be a potential curative treatment, as like CTLA-4 haploinsufficiency, only few patients with LRBA deficiency have been transplanted to date (41, 45, 55).

Slatter et al. described HCT in 8 patients with CTLA-4 haploinsufficiency. The diagnosis was made retrospectively in 7 of 8 patients who underwent HCT for life threatening treatment-refractory immune dysregulation (56). All received 10/10 HLA-matched unrelated donors following reduced intensity conditioning regimes. Six of the 8 patients are alive and well with donor chimerism ranging 85–100%. The one patient that was diagnosed pre-HCT was treated with abatacept but did not change the indication for HCT which was Non-Hodgkin lymphoma. In the cohort described by Schwab et al, 12 patients underwent HCT between 10 and 50 years of age, with indications for HCT including refractory cytopenia, enteropathy, lymphoproliferation and malignancy. Nine of 12 are alive, 3 of them beyond 5 years after HCT. Three of 12 received abatacept to control disease manifestations and all 3 survived post HCT (**Table 2**). Tesch et al. recently described the largest cohort of 76 patients with LRBA deficiency, 24 of whom underwent HCT. The median time between onset of disease and HCT was 7.4 years (0.4 —15.8 years). Pre-HCT disease burden and treatment response were assessed using a specialized scoring system, termed the Immunodeficiency and Dysregulation Activity score (IDDA). A favorable degree of disease remission was observed in the HCT cohort (70.6%) compared to patients who were treated on immunosuppressive therapy alone (11.6%) (45) (**Table 2**). Further, those treated with abatacept had the largest reduction in IDDA. Lower disease activity score at time of HCT correlated with higher survival (45). Despite the small cohort of patients, results from these studies are encouraging, supporting the idea that biologic therapy to control disease manifestations pre-HCT may improve survival post-HCT.

**Table 2:** Hematopoietic cell transplantation outcomes for CTLA-4 haploinsufficiency, LRBA deficiency, activated PI3Kd syndrome, and STAT1 and STAT3 Gain of Function.

Reference	N	Age at HCT, median (range)	Donor source	Stem cells	Conditioning regimen^a^	Serotherapy	Graft Failure	Grade II-IV aGVHD	Overall Survival	Event-free Survival^b^	Follow-up
**CTLA-4 Haploinsufficiency**											
Slatter et al. (56) and Schwab et al. (44)	12	15.5yrs (10-51 yrs)	MUD (7) MMUD 5	BM (6) PBSC (6)	RIC (12)	Alemtuzumab (7) ATG (4) 1 unknown	1/12 (8.3%)	7 (58%)	9/12 (75%)	8/12 (67%)	10mos (3mos-6.1 yrs)
**LRBA Deficiency**											
Tesch et al. (45)	24	10 years (1.3-24yrs)	MRD (13) MUD (8) MMUD (2) MMRD (1)	Not reported	Not reported	Unspecified serotherapy (22); including Alemtuzumab (2)	2/24 (8.3%)	5 (21%)	17/24 (70.8%)	17/24 (70.8%)	21mos (3-171 mos)
**Activated PI3Kδ Syndrome**											
Nademi et al. (57)	11	8 years (5-18yrs)	MRD (4) MUD (5) MMUD (2)	BM (3) PBSC (7) UCB (1)	MAC (7) RIC (4)	ATG (3) alemtuzumab (5)	None	4 (36%)	9/11 (81%)	7/11 (64%)	4 years (8mos-16yrs)
Okano et al. (58)	9	11 years (4-17yrs)	MMRD (4) MUD (3) MMUD (2)	BM (8) UCB (1)	MAC (1) RIC (8)	ATG (8)	2 (22%)	1 (11%)	7/9 (78%)	5/9 (56%)	Not reported
**STAT1 Gain of Function**											
Leiding et al. (59)	15	10 years (1-33yrs)	MRD (4) MUD (8) MMUD (3)	BM (10) PBSC (3) UCB (2)	MAC (7) RIC (7) None (1)	ATG (6) alemtuzumab (4)	7 (47%)	4 (27%)	6/15 (40%)	4/15 (27%)	surviving patients >1 year post-HCT
Kiykim et al. (60)	2	2 & 2.5 yrs	MUD (1) MRD (1)	BM (1) UCB (1)	MAC (1)	ATG (2)	1 (50%)	1 (50%)	1/2 (50%)	1/2 (50%)	24 mos
**STAT3 Gain of Function**											
Milner et al. (61)	2	11 & 16 yrs	MUD (1) MMUD (1)	Not reported	MAC (1) RIC (1)	alemtuzumab (2)	None	1 (50%)	1/2 (50%)	1 (50%)	No reported

MRD, matched related donor; MMRD, mismatched related donor; MUD, matched unrelated donor; MMUD, mismatched unrelated donor; BM, bone marrow; PBSC, peripheral blood stem cells; UCB, umbilical cord blood; MAC, myeloablative conditioning; RIC, reduced intensity conditioning; ATG, anti-thymocyte globulin; HCT, hematopoietic cell transplantation.

a Per CIBMTR pre-HCT preparative regimen guidelines. Note that reduced-toxicity conditioning regimens were classified as myeloablative.

b Where death, graft failure, recurrence of disease, or need for second HCT were considered events.

## Activated PI3K δ Syndrome

Activated phosphoinositide 3-kinase δ syndrome (APDS) is an inherited primary immunodeficiency due to heterozygous gain of function mutations (GOF) in *PIK3CD*, which encodes the catalytic subunit p110δ of phosphoinositide 3-kinase δ (PI3Kδ), or heterozygous loss of function mutations in *PIK3RI*, which encodes the regulatory subunit p85. PI3Kδ is a lipid kinase expressed predominantly in leukocytes, and mutations in either *PIK3CD* or *PIK3RI* result in hyperactivation of the AKT/mammalian target of rapamycin (mTOR) signaling pathway, which is critical role in regulating a diversity of immune responses (**Figure 1B**). Typical immunologic abnormalities include defects in immunoglobulin class switching resulting in hypogammaglobulinemia, often with elevated IgM levels, variable degrees of lymphopenia characterized by T cell senescence and skewing of CD8+ T cells to the effector phenotype, and impaired response to vaccines (46, 62). The clinical phenotype is also highly variable, but the most common clinical manifestations include peripheral lymphoproliferation, recurrent and severe infections, particularly of the respiratory tract, resulting in high rates of bronchiectasis, herpesvirus infections, and autoimmune and inflammatory disease, including autoimmune thyroid disease, autoimmune cytopenia and enteropathy, among others (46, 62). Patients are also at increased risk of lymphoma and other malignancy (46, 62).

Nademi et al. reported the first case series of 11 patients that underwent allogeneic HCT for APDS (57) (**Table 2**). Age at transplant ranged from 5 to 23 years. All patients had recurrent sinopulmonary infections, 4 had bronchiectasis, and 8 had generalized lymphadenopathy and hepatosplenomegaly. Viral infections and autoimmune and inflammatory disease were also common. Notably, five of the patients had received immunosuppression (steroids ± sirolimus) pre-transplant with inadequate disease control. Most patients received myeloablative conditioning (n=7, 64%) and serotherapy with alemtuzumab or antithymocyte globulin (n=8, 73%). Grafts were from a variety of sources. Ultimately, nine (81%) patients were alive at 8 months to 16 years follow-up, which is on par with survival rates reported for other primary immunodeficiencies. However, two patients had declining chimerism over time – one with recurrence of disease and one deemed to need repeat transplantation, which was refused by the patient’s family.

Okano et al. subsequently described 23 patients with APDS that included 9 patients who underwent allogeneic HCT (58) (**Table 2**). Age at transplant in this cohort ranged from 4 to 17 years. All patients who underwent HCT had a history of frequent and severe infections and/or lymphoproliferation inadequately controlled with immunosuppression. Conditioning was primarily with fludarabine-based reduced intensity conditioning regimens (n=8, 89%), and most patients received anti-thymocyte globulin (n=8, 89%). Notably, two patients experienced graft failure and received second transplants, and a third patient received a peripheral blood stem cell boost for poor graft function. Two other patients had mixed recipient and donor chimerism. Overall, post-transplant inflammatory complications were high, and two patients ultimately died.

Event-free survival (where an event is defined as death, graft failure, need for second transplant and disease relapse) was low in the two aforementioned series, possibly related to severity of disease and/or poor disease control at time of HCT. The high rate of graft failure and mixed chimerism reported by Okana et al. may be related to the fact that most patients received reduced intensity conditioning, suggesting that myeloablative conditioning may be necessary in patients with APDS to ensure full and durable donor stem cell engraftment. Better control of disease through the use of targeted therapy pre-transplant could potentially allow for the use of more intense conditioning regimens and better overall and event-free survival, but this is yet to be studied prospectively. Ideally, using a disease activity score such as the IDDA to standardize disease monitoring prospectively should occur.

Overactivation of PI3Kδ in patients with APDS results in hyperactivation of the Akt/mammalian target of rapamycin (mTOR) pathway. As such, sirolimus has been used to target mTOR in patients with APDS. In a cohort of 53 patients with APDS due to *PIK3CD* mutations described by Coulter et al, 6 patients received sirolimus for benign lymphoproliferation, and 5 of 6 (83%) patients had clinical improvement, although notably, sirolimus had to be discontinued in one patient due to intolerable side effects (46). Elkaim et al. reported on a cohort of 36 patients with APDS due to *PIK3R1* mutations that also included 6 patients that received sirolimus, and two of the patients had significant reduction in lymphoproliferation (47). Follow-up was too short in the other 4 patients to evaluate treatment efficacy.

Maccari et al. recently described disease evolution and response to sirolimus in APDS patients that included 26 patients treated with sirolimus (48) (**Table 1**). Like Coulter et al. and Elkaim et al. response rate in patients with lymphoproliferation was encouraging with 19 of 25 (76%) patients experiencing complete or partial remission of lymphoproliferation. However, sirolimus was less efficacious at treating colitis (6 of 15 or 40% with complete or partial remission) and AIC (5 of 14 or 36% with complete or partial remission). Overall, eight patients were on steroids when sirolimus was started, and steroids were completely discontinued in 7 patients and dose reduced in an eighth patient. Of note, sirolimus was stopped in 2 patients and had to be paused in 3 other patients due to side effects.

More recently, leniolisib, on oral small molecule inhibitor of the p100δ subunit of PI3K has been used as a more targeted therapy in patients with APDS (**Table 1**). Rao et al. published results from a preclinical, 12-week, open-label, within-patient, dose escalation study that included 6 patients (49). All 6 patients had lymphadenopathy and splenomegaly on CT or MRI at baseline, and all 6 patients had reduction in lymph node size (mean 40% reduction, range 13-65%) and spleen size (mean 39% reduction, range 26-57%) with leniolisib. Furthermore, all 6 patients reported improved energy levels and overall increased well-being. Physicians also reported less disease activity using a global assessment questionnaire. From an immunologic standpoint, leniolisib led to reduction in elevated transitional B cells and normalization of naïve B cells; IgM levels decreased; and frequencies of activated, exhausted, and senescent T cells decreased. Cytokine and chemokines abnormally elevated at baseline (TNF, IFNγ, CXCL13 and CXCL10) also decreased in all 6 patients. Furthermore, leniolisib was well tolerated at all doses on the 12-week dose-escalation study, and no significant adverse effects were reported on a 9-month extension study. Encouragingly, 5 of 6 patients opted to enroll in an open-label long-term extension study following completion of the 12-week treatment period.

Finally, the inhaled PI3Kδ, inhibitor, GSK2269557 or nemiralisib, is currently under investigation (NCT02593539) on a multi-center, non-randomized, open-label, uncontrolled, single group study to investigate the safety, pharmacokinetics and pharmacodynamics of nemiralisib in patients with APDS. Enrollment is complete, but results have not yet been reported. It will be interesting to see how patients with APDS respond to an inhaled PI3Kδ, inhibitor, especially those with pulmonary disease and bronchiectasis. Ultimately, while early results from studies targeted therapy in patients with APDS have been encouraging, long-term safety and efficacy are unknown.

## STAT1 and STAT3 Gain of Function

STAT1 and STAT3 are members of the signal transducer and activator of transcription (STAT) family of transcription factors that play key roles in a number of signaling pathways involved in the cellular response to interferons and a variety of other cytokines (**Figure 1C**). Patients with *STAT1* or *STAT3* GOF mutation have a broad spectrum of clinical manifestations. Patients with *STAT1* GOF mutation typically have augmented T helper type 1 (Th1) cells and impaired T helper type 17 (Th17) responses. Common clinical manifestations include chronic mucocutaneous candidiasis, recurrent infections with bacteria, dimorphic fungi, herpes viruses, and nontuberculous mycobacteria, and autoimmune disease, including hypothyroidism, type 1 diabetes, and autoimmune cytopenia (63). *STAT3* GOF mutation results in impairment of the regulatory T cell compartment, and patients characteristically suffer from lymphoproliferation, recurrent infections, and multisystem autoimmune disease, including type 1 diabetes, enteropathy and autoimmune cytopenia (61, 64). Short stature is also common (61, 64).

Published transplant outcomes for patients with *STAT1* GOF mutations have not been encouraging (**Table 2**). Leiding et al. reported the largest series of 15 patients with *STAT1* GOF mutation that underwent allogeneic HCT (59). Age at transplant ranged from 4 to 33 years. All 15 patients suffered from infections; 10 of 15 patients had autoimmune disease; and 2 patients had features consistent with HLH. About half (n=7, 47%) of patients received myeloablative conditioning, and the other half (n=7, 47%) received reduced intensity conditioning. One patient with a combined immune deficiency phenotype received no conditioning. Most (n=12, 80%) patients received grafts from HLA-matched donors. In general, post-transplant complications were frequent, and graft failure in particular was common – 1 patient had primary graft failure and 6 patients had secondary graft failure for an overall graft failure rate of 47%. Interestingly, graft failure was not associated with patient age, conditioning regimen or graft source. Ultimately, 5 of the 7 patients with graft failure died. Overall survival and event-free survival of the cohort as a whole were markedly low at only 40% and 27%, respectively. Age at HCT was the strongest predictor of overall survival, suggesting that patients fare better when they are transplanted before developing severe and/or refractory disease.

Kiykim et al. subsequently reported HCT outcomes for two patients with *STAT1* GOF mutation (60). Both patients received reduced-intensity conditioning, and grafts were from matched related and matched unrelated donors. HCT was successful in one patient with resolution of disease phenotype and correction of the enhanced STAT1 phosphorylation, dysregulated IFNγ production, and suppressed IL-17 response present at baseline. However, the second patient suffered from secondary graft failure and died from complications of HCT, again suggesting HCT for STAT1 GOF is complicated by high rates of graft failure.

The literature on HCT for patients with *STAT3* GOF mutation is more limited. Milner et al. reported two patients who underwent unrelated donor HCT; one patient received myeloablative conditioning and the other reduced intensity conditioning. One patient died from severe graft versus host disease (GVHD) and disseminated adenoviral infection. However, the other patient was a 16-year-old with a history of autoimmune cytopenia, hypothyroidism, enteropathy and recurrent pulmonary infections was alive and well at last follow-up with improved growth and resolution of autoimmunity (61) (**Table 2**). A recent review of the literature by Fabre et al. identified 5 published patients with *STAT3* GOF mutation that underwent allogeneic HCT (including the 2 previously reported by Milner et al), and disappointingly, only 1 of 5 patients survived (65). However, details of the patients’ clinical status and transplantation procedures were not provided, making it difficult to reach any conclusions regarding HCT for STAT3 GOF.

Ultimately, the immune dysregulation associated with *STAT1* and *STAT3* GOF mutations has been difficult to treat, and many patients require the use of multiple immunosuppressants. Poor clinical status and at time of transplantation is likely a primary factor behind the reported poor overall and event-free survival following HCT. Furthermore, the high rate of graft failure in patients with *STAT1* GOF mutation is thought to be related to elevated baseline IFNγ levels, where IFNγ has been shown to mediate rejection of hematopoietic stem cells in patients with graft failure (32, 66). As such, bridging therapy capable of normalizing IFNγ levels could potentially be beneficial for disease control and also engraftment post-transplant. That being said, prospective trials investigating this hypothesis are needed.

Upon cytokine receptor engagement and dimerization, janus kinase (JAK) proteins trans-phosphorylate, and the activated JAKs subsequently phosphorylate STAT transcription factors to mediate diverse immune effects (**Figure 1C**). As such, JAK inhibitors (jakinibs) such as ruxolitinib or tofacitinib have been used in recent years to target the STAT hyperresponsiveness in patients with STAT1 and STAT3 GOF, and there have been several case reports documenting the successful use of ruxolitinib in patients with STAT1 gain of function mutations with reversal of the clinical phenotype (67–71). Forbes et al. recently published on the use of jakinibs (ruxolitinib or tofacitinib) in 11 patients with STAT1 GOF and 6 patients with STAT3 GOF (50). Indications for jakinib therapy included autoimmune disease or immune dysregulation (n=16, 94%), immune suppression prior to HCT (n=1, 6%), HLH (n=1, 6%), and/or chronic progressive infection (n=8, 47%). An impressive 14 of 17 (82%) patients had significant clinical improvement, and chronic mucocutaneous candidiasis resolved in all patients with STAT1 GOF (**Table 1**). Furthermore, medication side effects were rare, and no patient had to be taken off therapy. In the case of STAT3 GOF patients received a combination of tocilizumab and jakinib therapy and 4 of 6 had resolution of disease manifestations.

The role of ruxolitinib or other jakinibs as a bridge to HCT is unknown. In the HCT cohort described by Leiding et al, one patient received ruxolitinib for 2 weeks before HCT, and this patient was one of the 4 patients that survived with no secondary graft failure, suggesting that ruxolitinib may be effective bridge to transplant (59). More recently, Kayaoglu et al. described a patient with STAT1 GOF who received ruxolitinib in preparation for HCT and demonstrated that baseline enhanced STAT1 phosphorylation and dysregulated gene expression both improved with ruxolitnib treatment and completely normalized after HCT (72). However, further studies expressly describing the use of jakinibs in patients with STAT1 and STAT3 GOF in preparation for transplant are needed.

## Conclusions

PIRDs are a newly recognized group of diseases dominated by immune mediated pathology. Treatment of PIRD related manifestations has led to major advances in the use of biologic modifiers altering the mechanism of disease and controlling disease manifestations. Despite this recent progress in precision biologics controlling PIRD related symptoms, substantial data are lacking. The appropriate patient to receive therapy and the appropriate dose, schedule, and safety profile for these medications are unknown. The long-term efficacy, ability of these agents to prevent disease related manifestations is unknown. Further, the use of targeted therapy is unlikely to eliminate the need for HCT for patients with severe disease, and at least in some PIRDs, there is an increased risk of malignancy mainly of the hematopoietic system, which is unlikely to be corrected through life-long pharmacological immunosuppression and thus could represent another indication to evaluate a patient for HCT early. Questions remain as to which patients should be considered for HCT, the optimal timing of HCT, and optimal HCT procedures, including identification of conditioning regimens associated with successful donor engraftment. It is also unclear what percent donor chimerism is necessary to correct the immune phenotype, including lymphoproliferation and risk of malignancy. Despite this lack of data it is clear that in HLH, patient condition pre-HCT directly impacts overall survival post-HCT. As is the case with HLH and now several cases of PIRD, precision based treatment of the patient leading to underlying disease control or remission lead to improved outcomes. Controlling the underlying PIRD inflammation and disease manifestations prior to HCT with precision therapy has the potential benefit to reduce risk of graft loss, reducing the incidence of GVHD, and improving overall survival and event free survival following HCT. The benefit of controlling the underlying disease, however, must be balanced around the timing of HCT and should not lead to substantial delays in definitive treatment. Although there are many unanswered questions regarding best treatments for PIRD and how the use of biologic modifiers are used to bridge HCT, precision based biologic modifiers seem to show benefit in controlling disease symptoms leading up to HCT. Further studies are necessary to answer these many questions about the utility in precision therapy for treatment of PIRD.

## Author Contributions

All authors listed have made a substantial, direct, and intellectual contribution to the work, and approved it for publication.

## Funding

JL and DC are supported in part by the Love McKinley Foundation.

## Conflict of Interest

JL - Consultant for Pharming (manufacturer of leniolisib) and Sobi (manufacturer of emapalumab).

The remaining authors declare that the research was conducted in the absence of any commercial or financial relationships that could be construed as a potential conflict of interest.
